# Retraction: Inguinal Hernia Containing an Inflamed Appendix: A Case of Amyand Hernia

**DOI:** 10.7759/cureus.r126

**Published:** 2024-01-25

**Authors:** Deena M Aldosari, Nourh K Alaboon, Mohammed Y Mojammami, Mohammed O Aqeeli, Omar A Aldhafeeri, Ali A Theban, Ammar Y Bafarat, Najd A Almutairi, Mohammed H Alotaibi, Ali M Humood, Emad Y Alqurashi, Abdulmajed A Alramih, Abdulgader A Mira, Abdulhakeem M Khan, Faisal Al-Hawaj

**Affiliations:** 1 College of Medicine, King Saud University, Riyadh, SAU; 2 College of Medicine, Princess Nourah Bint Abdul Rahman University, Riyadh, SAU; 3 College of Medicine, Jazan University, Jazan, SAU; 4 College of Medicine, King Abdulaziz University, Jeddah, SAU; 5 College of Medicine, Shandong University, Jinan, CHN; 6 General Practice, Ministry of Health, Riyadh, SAU; 7 College of Medicine, King Saud Bin Abdulaziz University for Health Sciences, Jeddah, SAU; 8 College of Medicine, Imam Abdulrahman Bin Faisal University, Dammam, SAU; 9 College of Medicine, Arabian Gulf University, Manama, BHR; 10 College of Medicine, Taif University, Taif, SAU; 11 College of Medicine, Dar Al Uloom University, Riyadh, SAU; 12 College of Medicine, Marmara University, Istanbul, TUR

The Editors-in-Chief have retracted this article. Concerns were raised regarding the identity of the authors on this article. Specifically, Faisal Alhaway and Malak Shammari have stated that they were added to this article without their knowledge or approval. The identity of the other authors could also not be verified. As the appropriate authorship of this work cannot be established, the Editors-in-Chief no longer have confidence in the results and conclusions of this article.

